# Plant-Based Potential in Diabetes Management: In Vitro Antioxidant, Wound-Healing, and Enzyme Inhibitory Activities of Southern Algarve Species

**DOI:** 10.3390/molecules30112432

**Published:** 2025-06-01

**Authors:** Isabel S. Carvalho, Cláudia Viegas, Marta Markiewicz, Agnieszka Galanty, Paweł Paśko, Lejsa Jakupović, Marijana Zovko Končić

**Affiliations:** 1 MED—Mediterranean Institute for Agriculture, Environment and Development, CHANGE—Global Change and Sustainability Institute, Food Science Laboratory, Faculdade de Ciências e Tecnologia, Universidade do Algarve, Campus de Gambelas, Ed. 8, 8005-139 Faro, Portugal; viegas.claudiasofia@gmail.com; 2 Center for Marine Sciences (CCMar), Universidade do Algarve, Campus de Gambelas, 8005-139 Faro, Portugal; 3 Department of Food Chemistry and Nutrition, Jagiellonian University Medical College, Medyczna 9, 30-688 Cracow, Poland; marta.markiewicz@doctoral.uj.edu.pl (M.M.); p.pasko@uj.edu.pl (P.P.); 4 Doctoral School of Medical and Health Sciences, Jagiellonian University Medical College, 16 Łazarza Str., 31-530 Cracow, Poland; 5 Department of Pharmacognosy, Jagiellonian University Medical College, Medyczna 9, 30-688 Cracow, Poland; agnieszka.galanty@uj.edu.pl; 6 Department of Pharmacognosy University of Zagreb Faculty of Pharmacy and Biochemistry, Ante Kovačića 1, 10000 Zagreb, Croatia; ljakupovic@pharma.hr (L.J.); mzovko@pharma.hr (M.Z.K.)

**Keywords:** diabetes mellitus, antioxidants, wound-healing, antidiabetic potential, medicinal plant

## Abstract

Type 2 diabetes mellitus (T2DM) is a chronic metabolic disorder characterized by impaired glucose regulation. This study evaluated the antioxidant and antidiabetic potential of aqueous extracts from four plant species from the southern Algarve: *Aristolochia baetica*, *Chelidonium majus*, *Dittrichia viscosa*, and *Lavandula viridis*, using non-cellular in vitro assays. HPLC/PDA was used to identify active compounds. Antioxidant activity was assessed by using TAA, FRAP, RP, and DPPH assays; antidiabetic potential through α-glucosidase and α-amylase inhibition; and wound healing relevance through elastase, collagenase, and lipoxygenase inhibition. *D. viscosa* showed the highest antioxidant activity (FRAP: 1132.99 ± 19.54 mg TE/g dw; DPPH IC_50_ = 25.85 ± 0.75 μg/mL) and total phenolic/flavonoid content, with a diverse profile including caffeic and chlorogenic acids, isoquercetin, and quercetin. It also exhibited potent α-glucosidase inhibition (IC_50_ = 0.61 ± 0.06 mg/mL), outperforming acarbose. *L. viridis* had the highest total phenolic content (39.04 mg/g), while *A. baetica* demonstrated the strongest anti-elastase, anti-collagenase, and lipoxygenase activity, suggesting wound-healing potential. *C. majus* showed the weakest effects. A strong correlation was observed between phenolic content and antioxidant/antidiabetic activity. These findings support further in vivo studies on *D. viscosa* and *A. baetica* for potential use in T2DM management and diabetic wound healing.

## 1. Introduction

Currently, type 2 diabetes mellitus (DM) is a chronic metabolic disorder that results either from insufficient insulin production and secretion by the pancreas or a combination of insulin resistance in peripheral tissues and inadequate insulin production to compensate for this resistance [1]. In both cases, if the disease is not properly managed, it leads to hyperglycemia (elevated blood glucose levels) and disrupts the metabolism of carbohydrates, lipids, and proteins. Over time, these metabolic disturbances cause progressive damage to various organs, particularly the endothelial membrane, affecting blood vessels, kidneys, and the nervous system. This damage can lead to serious complications, such as cardiovascular disease, renal disease, retinopathy/cataracts, and diabetic ulcers [2,3].

In addition to hyperglycemia, other risk factors—such as hyperlipidemia, hypertension, and oxidative stress—also contribute to the likelihood of these complications. The development and progression of type 2 DM, as well as its complications, are largely driven by the “toxic” effects of elevated glucose levels in circulation, alongside biochemical disruptions in lipid metabolism, oxidative stress caused by free radicals, chronic inflammation (especially at the endothelial level), hypoxia, and tissue ischemia, but also the impaired wound-healing process [3,4]. As a result, individuals with diabetes have a fourfold increased risk of developing coronary artery disease (such as myocardial infarction and angina) and a tenfold increased risk of developing peripheral vascular disease compared to non-diabetic individuals. This heightened risk translates into a three to fourfold increase in mortality from cardiovascular diseases among diabetic individuals [5,6].

In managing type 2 DM, reducing postprandial hyperglycemia is a critical therapeutic strategy. One effective approach is to decrease carbohydrate absorption following ingestion, which can be achieved by inhibiting the enzymes ɑ-amylase and ɑ-glucosidase. These enzymes are pivotal in the transformation of dietary carbohydrates into glucose, and their activity significantly impacts both the pathophysiology and management of type 2 DM [7,8]. Numerous plant extracts and natural products, many of which are derived from traditional medicinal plants, act through their capacity to inhibit ɑ-amylase and ɑ-glucosidase [9]. One of them is acarbose, originally isolated from *Actinoplanes* sp., a soil-dwelling actinomycete, and now a well-established synthetic antihyperglycemic agent. Acarbose functions through the competitive inhibition of these enzymes, thereby regulating glucose absorption in the intestine. Despite its effectiveness, acarbose is associated with adverse effects, such as abdominal pain, diarrhea, and flatulence [7,8,10]. Given these side effects, there is a pressing need to explore and analyze natural alternatives that offer similar mechanisms of action but with potentially fewer adverse effects. This study seeks to identify such alternatives by investigating the enzyme inhibition potential of various natural extracts.

Polyphenols, found in a variety of plant species, have shown potential in inhibiting digestive enzymes responsible for carbohydrate hydrolysis and absorption in the stomach and intestine. Their ability to bind to these enzymes suggests a role in managing postprandial hyperglycemia [11,12,13]. Historically, antioxidants from natural sources have been utilized, albeit empirically, for their health benefits [14]. Modern scientific research has validated these benefits, particularly in the context of diseases linked to oxidative stress, such as DM, and their applications extend to cosmetics as well [15,16]. The role of antioxidants, especially polyphenols, in diabetes prevention and management is well-documented. These compounds offer a range of beneficial effects, including enhancing insulin sensitivity, reducing oxidative stress, modulating inflammation, and improving glucose metabolism. When combined with healthy lifestyle habits, the inclusion of polyphenol-rich plant foods in a balanced diet offers a promising strategy for both the prevention and management of type 2 DM [15,17,18,19].

In this study, we aim to identify active compounds and investigate the antidiabetic, wound-healing, and antioxidant activities of aqueous extracts from plants native to the Algarve region of Portugal. Specifically, we will focus on four plant species: *Aristolochia baetica* L. (Aristolochiaceae), *Chelidonium majus* L. (Papaveraceae), *Dittrichia viscosa* L. (Asteraceae), and *Lavandula viridis* L. (Lamiaceae). These plant species are highly representative of the native flora of the Algarve region. They were selected for this study due to their richness in phenolic compounds and the limited documented references regarding their medicinal applications, particularly in the context of type 2 DM. Our research aims to explore their efficacy in both the prevention and management of this condition.

## 2. Results and Discussions

In this study, we evaluated the antioxidant and antidiabetic activities of four aqueous extracts from plants native to the Algarve region. To achieve our objectives, the aqueous extracts underwent several tests to assess their antioxidant properties, including Total Phenolic Content (TPC), Total Flavonoid Content (TFC), Total Antioxidant Activity (TAA), Ferric Reducing Antioxidant Power (FRAP), Reducing Power (RP), and DPPH radical scavenging activity. Additionally, inhibition assays for two key antidiabetic enzymes, ɑ-glucosidase and ɑ-amylase, as well as the enzymes elastase, collagenase, and lipoxygenase, related to diabetic wound healing. The content of the extracted plant material (*w/v*) and the efficiency of the extraction process (%) were initially determined. The results are summarized in Table 1. Table 2 summarizes the mean concentrations, along with standard deviations, of TPC, TFC, TAA, FRAP, and RP assays, as well as the IC_50_ values from the DPPH assay, for the various aqueous plant extracts analyzed. Table 2 also includes the percentage contribution of total flavonoid content to the total phenolic content—TFC/TPC (%).

The extraction results (Table 1) indicate that *D. viscosa* yielded the highest extract concentration (13.40 mg/mL) and extraction efficiency (16.75%), suggesting it is the most extractable species under the tested conditions, whereas *C. majus* showed the lowest values for both parameters, reflecting a lower recovery of compounds. Although some polyphenols are known to have limited water solubility, aqueous extraction was intentionally employed to replicate traditional preparation methods, such as 15 min infusions or decoctions, commonly used by local populations [18]. While this method may be less efficient for extracting certain compounds, it enhances the ethnopharmacological relevance of the study and offers valuable insight into the biological activities of the plants as traditionally consumed. Also, to maintain the study’s relevance, no additional purification steps were performed to remove ballast substances such as chlorophyll or cellulose, as the crude aqueous extracts were intended to reflect the traditional preparations used by local populations.

The data, summarized in Table 2, reveal significant differences in both total phenolic and flavonoid contents among the plant extracts, with statistical significance (*p* < 0.05). The TPC results show a range from 76.51 ± 3.84 to 477 ± 22.29 mg gallic acid equivalents per gram of dry weight. *D. viscosa* exhibited the highest phenolic content, with 477.10 ± 22.29 mg GAE/g, distinguishing it from the other plants. This extract also had the second highest total flavonoid content (22.87 ± 1.73 mg quercetin equivalents per gram of dry weight). In contrast, *C.majus* had the lowest phenolic content (76.51 ± 3.84 mg GAE/g).

For flavonoid content, *A. baetica* had the highest value (25.28 ± 0.57 mg QE/g dry weight), followed by *D. viscosa* and *L. viridis*, with *C. majus* again showing the lowest content (3.16 ± 1.11 mg QE/g dry weight).

When comparing the contribution of flavonoid content to the total phenolic content, Table 2 indicates that a high flavonoid content does not necessarily correlate with the highest phenolic values. This suggests that different extracts contain varying proportions of other phenolic compounds. Notably, *D. viscosa*—while having high total contents of both phenolics and flavonoids, shows only 4.79% of its phenolic compounds as flavonoids. Conversely, *A. baetica* has the highest proportion of flavonoid content relative to its total phenolic content, at 21.28%. These variations may be linked to taxonomic and ecological factors. Although the studied species belong to different botanical families, their phytochemical profiles may reflect adaptive responses to the environmental conditions of the Algarve, a region characterized by high UV exposure, drought periods, and nutrient-poor soils. Phenolic compounds, particularly flavonoids, are known to play protective roles against such stresses, and species like *D. viscosa* and *L. viridis*, with higher contents, may be better adapted to these harsh conditions [15,17].

Moreover, the results resonate with the traditional uses of these plants in local medicinal practices*. D. viscosa*, often used for its anti-inflammatory and antimicrobial properties, shows a phytochemical profile that supports these ethnopharmacological applications. Likewise, the rich flavonoid content in *A. baetica* may underpin its traditional use in treating circulatory and inflammatory disorders. In contrast, the comparatively low phenolic and flavonoid levels in *C. majus* may reflect its more specialized or less frequent traditional applications.

### 2.1. Quantification of Phenolic Compounds

HPLC/PDA analysis of aqueous extracts showed the presence of different phenolic compounds, including flavonoids such as rutoside and quercetin and isoquercetin and phenolic acids such as chlorogenic, isochlorogenic, caffeic, protocatechuic, and sinapic acids. Representative chromatograms of each extract are presented in the Supplementary Materials (Figure S1), and the detailed results are shown in Table 3. To our knowledge, *A. baetica* phenolics composition has not so far been examined and mentioned in the literature, and our results showed that it contained chlorogenic, caffeic acids, and rutoside. *C. majus* phytochemical composition is mentioned in the literature, mainly in the context of alkaloid compounds but also phenolic fractions [20], which we wanted to examine further. In the *C. majus* extract caffeic and chlorogenic acids, as well as rutoside and quercetin, were found. There are a few studies describing the bioactive components of *D. viscosa*. Similar to the study by Mrid et al. (2022) [21] we detected caffeic acid in our aqueous extract. However, unlike their results, we did not observe caffeoylquinic acid or its derivatives. This divergence may primarily stem from methodological differences: their study used a methanolic extract, which tends to extract a broader range of polyphenols compared to aqueous solvents. In contrast, our use of water as the extraction medium may have limited the solubility and recovery of certain less polar compounds. Another important factor contributing to the differences is the source of the plant material. In our study, aerial parts (leaves and stems) of *D. viscosa* were collected from wild populations growing spontaneously in the Algarve region of southern Portugal, specifically in the Barranco do Velho area. In contrast, Ben Mrid et al. [21] harvested *D. viscosa* leaves from the suburbs of Taza, Morocco. Geographic and environmental conditions—such as climate, altitude, soil composition, and seasonal timing—are well known to influence the accumulation of secondary metabolites. Moreover, the specific plant parts used (e.g., whole aerial parts vs. only leaves) may further affect the extracts’ chemical profile. Despite these differences, our findings are consistent with those reported by Pane et al. (2023) [22], who identified protocatechuic acid, chlorogenic acid, caffeic acid, and quercetin in *D. viscosa* extracts obtained from plants growing in the Cilento, Vallo di Diano, and Alburni National Park in southern Italy. This convergence reinforces the importance of these compounds as core bioactives in *D. viscosa*, regardless of environmental and methodological variation, and supports their relevance in the plants’ pharmacological potential.

While *L. viridis* has been studied primarily in the context of its essential oils and their biological activity, reports on its phenolic composition remain limited. Our results confirm the presence of caffeic acid, which is consistent with the previous literature [23,24]. In addition, we identified chlorogenic, isochlorogenic, protocatechuic, and sinapic acids in our aqueous extract. However, other studies have reported the presence of o-caffeoylquinic, fertaric, ferulic, and rosmarinic acids [24].

*D. viscosa* contained the largest variety of phenolic compounds (six out of eight recognized structures), but the plant with the highest content was *L. viridis* with the sum of all phenolic compounds being 39.04 mg/g. In comparison *D. viscosa*, *A. baetica*, and *C. majus* contained 11.78, 2.67, and 0.97 mg/g of phenolic compounds, respectively. These differences are likely due not only to the type of extraction solvent—as many previous studies used methanolic extracts, which tend to extract a broader spectrum of phenolic compounds—but also to other important variables, such as the plant part used, geographical origin, harvest time, and environmental conditions. All of these factors can significantly affect the qualitative and quantitative phenolic profile. In our case, we used the aerial parts of *L. viridis* collected from the Algarve region in southern Portugal, which may contribute to the observed variation. Therefore, the discrepancy cannot be attributed solely to the extraction solvent but rather to a combination of methodological and biological factors.

As previously mentioned, phenolic compounds are closely linked to the biological activity of plant extracts, particularly their antioxidant properties [14,16]. The total phenolic content (TPC) was first evaluated, followed by the quantification of total flavonoid content (TFC), as flavonoids represent a major subclass of phenolic compounds with recognized antioxidant properties.

### 2.2. Evaluation of In Vitro Antioxidant Capacity

The Total Antioxidant Activity (TAA) method measures the ability of compounds to reduce Molybdenum (VI) to Molybdenum (V), while the Ferric Reducing Antioxidant Power (FRAP) method evaluates the reduction of the TPTZ complex from ferric (Fe^3+^) to ferrous (Fe^2+^) ions. Similarly, the Reducing Power (RP) method assesses the reduction of Fe^3+^ to Fe^2+^ in the presence of a ferrocyanide complex. All three methods are the indicators of electron transfer activity and complement each other in assessing the antioxidant potential of the extracts [25].

Table 2 presents the statistical analysis for the antioxidant assays (TAA, FRAP, and RP), revealing statistically significant differences (*p* < 0.05) in antioxidant activity among the plant extracts. *D. viscosa* emerged as the most effective, demonstrating superior results across all three methods. This extract exhibited high total antioxidant activity, with a capacity to reduce Mo^6+^ to Mo^5+^ (704.54 ± 26.21 mg TAA/g dry weight), reduce the Fe-TPTZ (III) complex to Fe-TPTZ (II) using the FRAP method (1132.99 ± 19.54 mg TE/g dry weight), and convert Fe^3+^ to Fe^2+^ using the RP method (623.35 ± 24.02 mg TE/g dry weight). These findings are consistent with the high phenolic content reported in the TPC analysis, suggesting that the antioxidant activity of *D. viscosa* is largely due to its phenolic compounds and their electron-transfer mechanisms.

In this study, Pearson’s test was applied to examine correlations between all the analyzed variables, and the results are summarized in Table 4. Pearson’s Correlation is a statistical method used to assess the strength and direction of the linear relationship between continuous quantitative variables, providing insight into how they vary together [26,27]. TPC was significantly correlated with TAA, FRAP, and RP, while TAA highly significantly correlated with both FRAP and RP. FRAP and RP also exhibited a highly significant correlation. This indicates that phenolic compounds play a pivotal role in the antioxidant activity of the analyzed herbal extracts [14,15,16]. This is not surprising because the Folin–Ciocalteu test and the Ferric Reducing Antioxidant Power (FRAP) test are based on the transfer of one electron, similarly to the TAA and RP test. While electron transfer is a key mechanism of antioxidant activity, other mechanisms may also contribute [25]. Nevertheless, the high phenolic content and strong electron transfer capacity of *D. viscosa* highlight its potential as an effective antioxidant.

Following *D. viscosa*, *L. viridis* showed middle values of antioxidant capacity compared to the other plant extracts, with results of 561.30 ± 36.28 mg AAE/g dry weight using the TAA method, 785.45 ± 1.11 mg TE/g dry weight using the FRAP method, and 438.89 ± 17.94 mg TE/g dry weight using the RP method. This moderate activity aligns with its intermediate phenolic content.

In contrast, *A. baetica* and *C. majus* exhibited lower antioxidant activities, consistent with their lower phenolic contents. Specifically, *C. majus* had significantly lower values in both the FRAP (130.23 ± 2.53 mg TE/g dry weight) and RP (61.86 ± 2.77 mg TE/g dry weight) assays, indicating a limited capacity for ferric ion reduction compared to other extracts. Overall, the effectiveness of the aqueous extracts in terms of antioxidant activity, as determined by these methods, follows this descending order: *D. viscosa* > *L. viridis* > *A. baetica* > *C. majus*.

In Table 2 are the concentrations of aqueous extracts needed to scavenge 50% of DPPH free radicals (IC_50_). Statistical analysis indicates significant differences (*p* < 0.05) in DPPH scavenging activities among the studied plants. *D. viscosa* and *L. viridis* exhibit similar DPPH radical scavenging capacities, while *C. majus* shows markedly lower efficacy with an IC_50_ of 125.01 ± 8.84 µg/mL, indicating a poorer ability to stabilize free radicals compared to *D. viscosa* (25.85 ± 0.75 µg/mL) and *L. viridis* (29.52 ± 0.75 µg/mL). These findings are consistent with the low phenolic and flavonoid contents observed in *C. majus* and its reduced performance in the FRAP and RP assays. *D. viscosa* and *L. viridis* again demonstrate high antioxidant capacity, aligning with their strong performance in previous antioxidant assays. *A. baetica* shows a moderate DPPH scavenging ability with an IC_50_ of 44.93 ± 3.26 µg/mL, suggesting reasonable antioxidant potential. In summary, the ranking of the studied plants based on their DPPH free radical scavenging capacity is the same as for the antioxidant activity methods.

### 2.3. Evaluation of Antidiabetic Activity in Vitro: Inhibitory Activity of α-Glucosidase and α-Amylase

To assess the potential antidiabetic activity of the aqueous extracts from the examined plants, we evaluated their capacity to inhibit α-glucosidase and α-amylase, the enzymes which play a crucial role in carbohydrate digestion and glucose absorption. Prolonged exposure to high postprandial glucose levels can adversely affect pancreatic β-cells, contributing to their progressive dysfunction and, ultimately, to type 2 diabetes [28,29].

**Figure 1:**
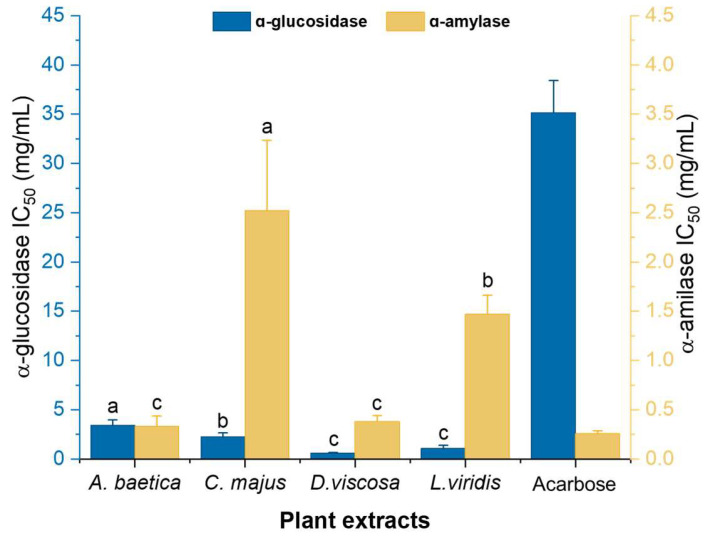
Inhibitory activity of ɑ-glucosidase and ɑ-amylase enzymes expressed as IC_50_ (mg/mL) of the four aqueous extracts of the studied. All results are expressed as the mean IC_50_ values ± SD (for n = 3). Different letters in the same enzyme test for the various samples (comparison of different samples in the same method) indicate that the samples have statistically significant differences determined by Games–Howell post-hoc test following one-way ANOVA (with *p* < 0.05). The standard inhibitor, acarbose, was included as a reference but was not subjected to statistical comparison and is therefore not assigned a superscript letter.

All four plant extracts demonstrated superior α-glucosidase inhibition compared to acarbose, a widely used antidiabetic drug with an IC_50_ of 3.52 mg/mL (Figure 1). Statistical analysis revealed significant differences (*p* < 0.05) in α-glucosidase inhibition among *A. baetica*, *C. majus*, *D. viscosa*, and *L. viridis*. Notably, *D. viscosa* and *L. viridis* exhibited similar α-glucosidase inhibitory activities. For α-amylase inhibition, significant differences were observed (*p* < 0.05) between *A. baetica*, *C. majus*, and *L. viridis*, as well as between *D. viscosa*, *C. majus*, and *L. viridis*. However, no significant difference was found between *A. baetica* and *D. viscosa*, suggesting comparable α-amylase inhibitory activity for these two plants (Figure 1).

Among the plants, *A. baetica* showed the highest α-amylase inhibitory activity with an IC_50_ of 0.33 ± 0.10 mg/mL and the lowest α-glucosidase inhibition with an IC_50_ of 3.44 ± 0.54 mg/mL. This pattern is similar to that of acarbose. The high flavonoid content in *A. baetica* (Table 2) likely contributes to its α-amylase inhibitory activity, supported by a significant negative correlation (*r* = −0.9799) between the flavonoid content and IC_50_ value of α-amylase inhibition (Table 4). *D. viscosa* excelled in α-glucosidase inhibition with an IC_50_ of 0.61 ± 0.06 mg/mL and it had the second-best α-amylase inhibitory activity (IC_50_ = 0.38 ± 0.06 mg/mL). This plant also showed the highest antioxidant capacity in TAA, FRAP, and RP assays. *L. viridis* exhibited intermediate antidiabetic activity with IC_50_ values of 1.07 ± 0.31 mg/mL for α-glucosidase and 1.47 ± 0.19 mg/mL for α-amylase. Its performance aligns with its intermediate phenolic content and antioxidant capacity. *C. majus* demonstrated the weakest antidiabetic activity among the plants studied, with IC_50_ values of 2.22 ± 0.43 mg/mL for α-glucosidase and 2.52 ± 0.71 mg/mL for α-amylase. This is consistent with its low phenolic and flavonoid content. Nevertheless, *C. majus* was more effective than *Morinda lucida*, a medicinal plant used in West Africa for diabetes treatment, which showed similar IC_50_ values for α-glucosidase and α-amylase in comparable studies [30].

The overall effectiveness of the plant extracts in inhibiting α-glucosidase and α-amylase can be ranked as follows: α-Glucosidase inhibition: *D. viscosa* > *L. viridis* > *C. majus* > *A. baetica*; α-amylase inhibition: *A. baetica* > *D. viscosa* > *L. viridis* > *C. majus* (Figure 1). Acarbose, as a positive control, was found to inhibit α-amylase more effectively than α-glucosidase, aligning with findings from Mabotja et al. [31]. The plant extracts generally exhibited greater α-glucosidase inhibitory activity compared to acarbose, reinforcing the notion that the plant compounds possess high antidiabetic potential. In conclusion, the plant extracts studied exhibit significant antidiabetic activity, with variations in their effectiveness against α-glucosidase and α-amylase. This supports the existing literature suggesting that phenolic compounds, particularly flavonoids, contribute to the inhibition of these digestive enzymes, thereby aiding in diabetes management. The plant species evaluated in this study have a history of traditional use in the Algarve region for managing digestive conditions, although scientific evidence supporting their efficacy remains limited. By investigating their phenolic composition and enzyme inhibitory potential, our study aims to bridge traditional knowledge with experimental validation and justify their selection over other species [8,11,13,32]. And, although the findings obtained in this study are promising, they should be interpreted with caution, as further validation through cell-based and in vivo studies is essential to confirm their biological relevance and potential therapeutic application.

### 2.4. Wound Healing Potential: Inhibitory Activity on Elastase, Collagenase, and Lipoxygenase

**Figure 2:**
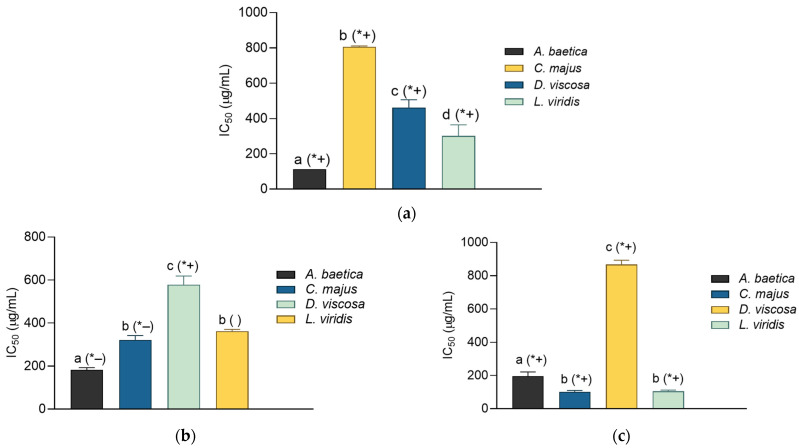
Inhibition of elastase (**a**), collagenase (**b**), and lipoxygenase (**c**) by the plant extracts (shown as IC_50_ values ± SD). Different lowercase letters indicate statistically significant difference between extracts, while the (*+), (*−), and ( ) signs indicate statistically different higher, statistically different lower, and statistically non-different IC_50_ value than the IC_50_ value of the standard inhibitor, respectively (*p* < 0.05). The employed standard inhibitors are as follows: ursolic acid (anti-elastase IC_50_ = 26.57 ± 0.37 μg/mL) (**a**), gallic acid (anti-collagenase IC_50_ = 376.56 ± 7.92 go/mL) (**b**), and nordihydroguaiaretic acid (anti- lipoxygenase IC_50_ = 8.21 ± 0.12 μg/mL) (**c**).

In this work the anti-elastase, anti-collagenase, and anti-LOX activity of the extracts was investigated. As may be observed in Figure 2a, all the extracts displayed an observable influence on elastase. However, none among them reached the activity of the standard inhibitor, ursolic acid (IC_50_ = 26.57 ± 0.37 µg/mL). Among the extracts, the most active was the one prepared from *A. baetica*, followed by *L. viridis*. The collagenase-inhibiting properties of the extracts are presented in Figure 2b. Most extracts displayed an excellent anti-collagenase activity with IC_50_ values that were lower (*A. baetica*, *C. majus*) or did not differ (*L. viridis*) statistically from that of the employed standard, gallic acid (IC_50_ value was 376.56 ± 7.92). The most active among the investigated samples was again *A. baetica* extract, closely followed by *C. majus* and *L. viridis*. The anti-LOX activity of the extract (Figure 2c), while being relatively modest in comparison with NDGA (IC_50_ = 8.21 ± 0.12 µg/mL), was still well-pronounced. The best activity in this assay was observed with *L. viridis* and *C. majus*, closely followed by *A. baetica*.

Together with the growing incidence of diabetes and the increasing number of elderly individuals, diabetic ulcers, a severe complication of diabetes mellitus, are among most prevalent chronic wounds in developed nations. While the options for the effective treatment of diabetic ulcers and other chronic wounds remain limited, the research on the agents that could promote their healing is increasing [33]. This also includes research on traditional medicines. Plant natural products, especially polysaccharides, flavonoids, and other polyphenols, have been extensively studied in treatment of diabetic ulcers where they have shown promising activity [34,35].

Collagen and elastin are essential proteins that form the primary structure of the extracellular matrix (ECM) in the skin, and as such, their restoration is an essential part of the wound-healing process. Collagen is responsible for tensile strength, while elastin fibers enhance the skin’s ability to stretch and return to its original shape [36]. Consequently, the combination of collagen and elastin fibers is crucial for maintaining the skin’s overall strength and mechanical characteristics, as well as the skin’s hydration [37,38]. Skin proteases, as well as those formed by the bacteria that may infect the wound, significantly affect wound healing. Elastases play an important role in the delayed healing of chronic wounds, including diabetic ulcers. Bacterial elastases have been identified as significant contributors to the wound’s tissue degradation and the delayed healing of infected wounds [39]. High levels of host’s neutrophil elastase were found to be associated with infection, while serum elastase is related to delayed healing and even worsening of the diabetic wounds [40]. Collagenases, the enzymes that break down collagen, belong to a larger group of matrix metalloproteinases (MMPs). MMPs are partly induced by chronic inflammation that occurs within the organism of diabetic patients. Elevated levels of proinflammatory cytokines stimulate fibroblasts to generate an excessive amount of collagenase and other MMPs, resulting in the breakdown of both functional and non-functional collagen fibers in ECM [41,42]. Thus, it is crucial to regulate MMPs’ activity during wound healing. This might be performed either by the direct influence on the MMPs or through the management of inflammation, such as by inhibition of the LOX enzyme. It has been found that impaired wound healing in type 1 diabetes is dependent on 5-lipoxygenase products such as leukotrienes [43].

The pronounced activity of the *A. baetica* and *L. viridis* extract in the performed assays may be related to their high flavonoid content, as well as the TFC/TPC ratio. Namely, *A. baetica* had the highest values of TFC and TFC/TPC among the investigated extracts, followed by the *L. viridis* extract. Flavonoids and other polyphenolic compounds have a high potential for the inhibition of elastase. For example, the kaempferol derivative, kaempferol-3-*O*-robinobioside, was a strong inhibitor of elastase and collagenase, both in silico and in vitro [44]. Hesperidin, its aglycone hesperetin, as well as its sugar components rutinose and rhamnose, are also notable elastase inhibitors [45]. Finally, phloretin, a polyphenol belonging to the dihydrochalcone type of flavonoids, has been shown to inhibit elastase and collagenase activity, as well as to suppress inflammatory processes by inhibiting the 12-*O*-tetradecanoylphorbol 13-acetate-induced expression of cyclooxygenase 2, a critical molecular target of many anti-inflammatory agents [46]. Other flavonoids, such as quercetin, apigenin, and kaempferol, were found to display strong anti-inflammatory activity by inhibiting numerous pro-inflammatory enzymes, including LOX [47,48]. Thus, further investigations, preferably followed by the isolation and determination of the activity of the individual components, are highly desired in order to elucidate the exact constituents and their roles in the overall activity. This approach will allow a better understanding of the mechanisms underlying the observed bioactivities and will be essential for the rational development of future plant-derived therapeutic agents.

## 3. Materials and Methods

### 3.1. Chemicals

The following chemicals were used: ferric chloride (Merck, Darmstadt, Germany); Folin–Ciocalteau reagent (Merck); allic acid (Fluka Chemie GmbH, Buchs, Switzerland); glacial acetic acid (BDH-Prolabo, Fontenay-sous-Bois, France); hydrochloric acid (Riedel-de Haën—Seelze, Germany); sodium acetate (Merck); Sodium carbonate (Merck); trichloroacetic acid (BDH-Prolabo); 2,4,6-tripyridyl-2- triazine (TPTZ) (Sigma Aldrich, St. Louis, MO, USA); Trolox; *N*-succinyl-(Ala)_3_-nitroanilide (Sigma Aldrich), ninhydrin, collagenase, elastase (Sigma Aldrich), acetonitryl (Merck), and formic acid (Merck). The α-amylases from porcine pancreas (TYPE VI-B) were purchased from Sigma-Aldrich, Gillingham, UK while the α-glucosidase used was obtained from yeast and purchased from Megazyme, Bray, Ireland. All α-amylases were in lyophilized powder form while α-glucosidase was in an ammonium-sulphate buffer suspension. The standards for HPLC/PDA analysis were purchased from Fluka Chemie GmbH, Buchs, Switzerland. All the reagents were of an analytical grade.

### 3.2. Samples

The aerial parts (leaves and stems) of *Aristolochia baetica* L., *Chelidonium majus* L., *Dittrichia viscosa* L., and *Lavandula virilis* L. were collected (Table 5) from the spontaneous flora of the Algarve region, Portugal, following identification by the botanists Rosa Pinto and Manuela David. Dried plant material was deposited as authenticated vouchers in the Herbarium of the University of Algarve (acronym ALGU), with the accession numbers 14531/ALGU, 14492/ALGU, 14541/ALGU, and 14543/ALUG, respectively.

### 3.3. Preparation of Samples

The plants were dried in boxes that allow aeration, at room temperature in the dark, and were reduced to powder by grinding and homogenizing using a cooking mill. Infusions (of 1.5 g dry weight—only the thicker roots and stems were not used) were made in a volume of 30 mL of distilled water (*w*/*v*) at a temperature of 95 °C for 15 min and with constant agitation of 200 rpm. The extraction yield (%) was calculated by comparing the mass of the substance obtained through extraction to the initial mass of the material used. Each extract was centrifuged at 5000 rpm for 15 min. The supernatant was recovered and kept at −20 °C until further analysis to avoid the compounds’ degradation. In parallel, 1 mL of each extract was placed in Eppendorf tubes and dried in the oven at 50 °C for 72 h to determine the dry residue per mL.

### 3.4. HPLC/PDA Analysis of Phenolic Compounds

The quantitative analysis of phenolic compounds was performed on the plants’ extracts with the use of the Dionex HPLC system, with a PDA 100 UV-VIS detector and a Hypersil Gold (C-18) column (5 μm, 250 × 4.6 mm, Thermo EC, Waltham, MA, USA), as previously described [49]. Briefly, the mobile phase consisted of (a) 1% formic acid and (b) acetonitrile, in gradient mode 5–60% B over 60 min, with the flow rate 1 mL/min and column temperature 20 °C. The concentration of each flavonoid and phenolic acid was calculated from the peak areas in relation to standard five-point calibration curves, generated using reference standards in the range of 0.0625–1 mg/mL. All the experiments were carried out in triplicate, and the data were reported as the mean values (mg/g of dried plant). The identification of individual phenolic acids and flavonoids in plant extracts was based on the comparisons of the retention times and UV spectra with authentic reference standards, which included p-coumaric acid, cinnamic acid, gallic acid, ferulic acid, vanillic acid, chlorogenic acid, sinapic acid, protocatechuic acid, gentisic acid, syringic acid, caffeic acid, quercetin, isoquercetin, and rutoside.

### 3.5. Analytical Methods

In all methods the T70 + UV/Vis Spectrometer, PG Instruments Ltd. (Wibtoft, UK) spectrophotometer was used as the signal measurement equipment in the form of absorbance and its computer system; the results are expressed in relation to the dry weight of the plant (mg/g) and in relation to the concentration in the extract (mg/mL); all of the experiments were conducted in triplicate.

### 3.6. Total Phenolic Content (TPC)

The total phenolic content of the extracts was evaluated using Folin–Ciocalteu reagent according to the method previously described by Singleton and Rossi [50]. The extracts (50 µL) were mixed with 125 µL of Folin–Ciocalteu’s phenol reagent (0.2 N), and 100 µL of 7.5% saturated sodium carbonate solution were left incubated for 1 h (in the dark) at room temperature. The absorbance was measured at 765 nm and the total phenol content was expressed as mg gallic acid equivalents (GAE) per g of dried plant after a calibration curve was obtained with diverse concentrations of gallic acid (0.001–1 mg/mL).

### 3.7. Total Flavonoid Content (TFC)

The total flavonoid content in the extracts was determined according to the method described by Lamaison and Carnat [51,52]. A solution made by 100 µL of methanolic AlCl_3_·6H_2_O 20% and 100 µL of each extract was left to stand for 1 h (in the dark) at room temperature; after that, the absorbance was read at 420 nm. The total flavonoid content was expressed as mg quercetin equivalents (QE) per g of dried plant after a calibration curve was obtained with diverse concentrations of quercetin (0.002–1 mg/mL).

### 3.8. Total Antioxidant Activity (TAA)

The total antioxidant activity (TAA) of the extracts was determined using a spectrophotometer method previously described by Prieto et al. [25]. Briefly, 100 µL of each extract was mixed with 1.0 mL of reagent solution (0.6 M sulfuric acid, 28 mM sodium phosphate, and 4 mM ammonium molybdate). The mixture was then incubated at 95 °C for 90 min in a water bath; after that, the absorbance was read at 695 nm. The total antioxidant activity was expressed as mg ascorbic acid equivalents (AAE) per g of dried plant after a calibration curve was obtained with diverse concentrations of quercetin (0.002–1 mg/mL).

### 3.9. Reducing Power (RP)

The reducing power was determined by the spectrophotometric method previously described by Oyaizu [53]. Briefly, 200 µL of properly diluted extract in 500 µL of sodium phosphate buffer (0.2 M, pH 6.6) was mixed with 500 µL potassium ferricyanide (1%) and the mixture was incubated at 50 C for 20 min. About 500 µL of trichloroacetic acid (10%) was then added and the mixture was centrifuged at 6500× g for 10 min. About 500 µL of supernatant was then mixed with 500 µL of distilled water and 100 µL of ferric chloride (0.1%) and the absorbance was read at 700 nm. The reducing power was expressed as mg Trolox equivalents (TE) per g of dried plant after a calibration curve was obtained with diverse concentrations of Trolox (0.002–1 mg/mL).

### 3.10. Ferric-Reducing Antioxidant Power (FRAP)

The ferric-reducing antioxidant power was determined by the spectrophotometric method previously described by Benzie and Strain [54]. Three stock solutions were prepared: a 300 mM acetate buffer (3.1 g sodium acetate trihydrate and 16 mL acetic acid for each liter of solution), pH = 3.6, a 10 mM TPTZ solution in 40 mM HCl, and a 20 mM ferric chloride hexahydrate solution. The working solution was prepared by mixing 25 mL of acetate buffer, 2.5 mL of TPTZ solution, and 2.5 mL of ferric chloride hexahydrate solution. This working solution was then heated to 37 °C. A 75 mL aliquot of properly diluted extract was mixed with 1.425 mL of the working solution. The absorbance was read at 593 nm 30 min after mixing. The ferric-reducing antioxidant power was expressed as mg Trolox equivalents (TE) per g of dried plant after a calibration curve was obtained with diverse concentrations of Trolox (0.002–1 mg/mL).

### 3.11. DPPH Radical Scavenging Capacity (DPPH)

The DPPH free-radical scavenging activity was assessed as described by Blois’s (1958) [55] method with slight modifications. Briefly, 1.0 mL of a 0.16 mM DPPH solution was added to the test tube containing 1.0 mL of properly diluted extract in different concentrations. The mixture was then vortexed for 1 min at 800 rpm and was kept in the dark for 30 min at room temperature. After this incubation time, the absorbance was read at a wavelength of 517 nm. Diverse extract concentrations were submitted to this procedure, and a graph of inhibition percentages versus extract concentration was made and the IC_50_ values were determined; these values are defined as the sample concentration providing 50% inhibition. For the evaluation of inhibition percentage (Inhibition %), the following formula was used:

(1) $$Inhibition\;\%=\frac{\left(A_{0}-A_{1}\right)}{A_{0}}\times 100$$

where A_0_ is the absorbance of the control (water instead of the plants extracts) and A_1_ is the absorbance of the sample. The same procedure was performed for the positive control, which was butylated hydroxytoluene (BHT) in a concentration range of 0.03–1 mg/mL.

### 3.12. α-Amylase Inhibition

Three α-amylases from different origins were tested: *Aspergillus oryzae*, porcine pancreas, and human saliva. Inhibition was determined following the method described by Sancheti et al. [56], with slight modifications. Briefly, 100 μL of a 2% starch solution were mixed with 50 μL of extract and incubated for 10 min at 20 °C. After incubation, 100 μL of 2 U/mL of α-amylase enzyme (prepared in pH 6.9 0.02 M and 0.0067 M NaCl phosphate buffer) were added and the obtained mixture was left to react for 5 min at 20 °C. After adding 100 μL of color reagent (sodium potassium tartarate and NaOH solution mixed with 96 mM 3,5-dinitrosalicylic acid) the mixture was incubated for 15 min at 95 °C. After cooling down, 900 μL of distilled water were added and the absorbance was read at 540 nm. The activities were presented as IC_50_ values and were determined as reported for the antioxidant activity. Acarbose was used as a positive control in the range of 0.002–1 mg/mL and was submitted to the same experimental conditions as the plant extracts.

### 3.13. α-Glucosidase Inhibition

A α-Glucosidase inhibition assay was carried out according to Li et al. [57], with some modifications. Briefly, 70 μL of the plant extracts were mixed with 50 μL of yeast α-glucosidase (2.4 U/mL) prepared in phosphate buffer (100 mM; pH = 6.8) and they were incubated at 37 °C for 10 min. After this period, 100 μL of a solution of *p*-nitrophenyl-β-D-glucopyranoside (PNPG) 5 mM was added to the same phosphate buffer. The reaction solution was incubated at room temperature for 30 min, and after this period, 80 μL of sodium carbonate solution (0.4 mM) was added to stop the reaction. The absorbance reading was performed at 405 nm. The activities were presented as IC_50_ values and determined as reported for the antioxidant activity. Acarbose was used as a positive control in the range of 0.002–1 mg/mL and was submitted to the same experimental conditions of the plant extracts.

### 3.14. Elastase Inhibition

The elastase inhibitory activity [58] was determined by preparing 100 μL of the extract solution in 0.1 M of Tris-HCl buffer (pH 8.0). The prepared solution was combined with the solution of elastase (25 µL, 0.05 mg/mL). Following a 5 min incubation at room temperature, 70 µL of a phosphate buffer saline solution of SANA (0.41 mg/mL) was added. After an additional 40 min, the resulting absorbance was measured at 410 nm (FLUOstar Omega, BMG Labtech, Ortenberg, Germany). The elastase inhibitory activity (ELAIn) was calculated as follows:

(2) $$ELAIn(\%)=\frac{\left(A_{0}-A_{s}\right)}{A_{0}}\times 100$$

where A_0_ is the absorbance of the negative control and A_s_ is the absorbance of the solution containing the respective extract. Ursolic acid (UA) was applied as the standard elastase inhibitor. The IC_50_ values were calculated as the concentration of the extract that inhibited 50% of elastase activity.

### 3.15. Collagenase Inhibition

For collagenase inhibition [58], 40 μL of the extract solution was added to the Tris-HCl buffer (0.1 M, pH 7.5), containing 5 mM CaCl_2_ and 1 µM ZnCl_2_. To the mixture, 20 µL of 1 mg/mL collagenase solution was added to the same buffer. After 5 min, 40 µL of 3.44 mg/mL gelatin solution was added to the same buffer. After an additional 40 min at 37 °C, stop reagent, containing 12% (*w*/*v*) PEG 6000, 25 mM EDTA, as well as 90 µL of the ninhydrin reagent (prepared by mixing SnCl_2_ solution (80 mg of SnCl_2_ × _2_H_2_O) dissolved in 50 mL of 0.2 M and pH 5.0 citrate buffer with an equal volume of ninhydrin solution prepared by dissolving 0.5 g of ninhydrin in 10 mL of DMSO) was added. The reaction mixture was incubated for an additional 15 min at 80 °C, and the absorbance was measured at 570 nm (FLUOstar Omega, BMG Labtech, Ortenberg, Germany). The inhibition of collagenase (COLIn) was calculated as follows:

(3) $$COLIn\;(\%)=\frac{\left(A_{0}-A_{s}\right)}{A_{0}}\times 100$$

where A_0_ is the absorbance of the negative control, and A_s_ is the absorbance of the solution containing the respective extract. Gallic acid (GA) was applied as the standard collagenase inhibitor. The IC_50_ values were calculated as the concentration of the extract that inhibited 50% of collagenase activity.

### 3.16. Lipoxygenase Inhibition

Lipoxygenase (LOX) inhibitory activity [59] was determined spectrophotometrically. Extracts (50 µL) or water (negative control) were mixed with phosphate buffer (150 μL, pH 8, 100 μM) and soybean LOX (20 μL), whereupon linoleic acid (30 μL) was added to initiate a reaction. After 10 min at 25 °C the absorbance at 234 nm was measured. The LOX inhibitory activity (LOXIn) was calculated as follows:

(4) $$LOXIn\;(\%)=\frac{\left(A_{0}-A_{s}\right)}{A_{0}}\times 100$$

where A_0_ is the absorbance of the negative control, and A_s_ is the absorbance of the solution containing the respective extract. Nordihydroguaiaretic acid (NDGA) was applied as the standard LOX inhibitor. The IC_50_ values were calculated as the concentration of the extract that inhibited 50% of LOX activity.

### 3.17. Statistical Analysis

All results are expressed in average ± standard deviation, and the results are from the analysis of three sample replicas (n = 3). The results were analyzed for statistical differences using the one-way ANOVA test with LSD or Games–Howel post-hoc tests after checking for the homogeneity of variances using Levene’s test in SPSS 22. Pearson linear correlation coefficients (r) were determined to analyze the correlations between the variables, which measured the direction and magnitude of the correlation between the assays. For anti-elastase, anti-collagenase, and anti-LOX assays, statistical comparisons were performed using one-way ANOVA followed by either Dunnett (comparisons of the individual extracts with the controls) or Tukey (for comparisons between the extracts) post-hoc tests.

## 4. Conclusions

The plants evaluated in this study, due to their bioactive compounds, have long been valued for their potential benefits in diabetes management. These compounds, especially those involved in carbohydrate metabolism, have been traditionally utilized by various populations, based on empirical knowledge. This study focused on evaluating the antioxidant capacity and antidiabetic activity of aqueous extracts from four plant species native to the Algarve region through in vitro analysis. Among the tested plants, *D. viscosa* displayed the most pronounced antioxidant activity and inhibition of the enzymes crucial for carbohydrate metabolism. On the other hand, the most promising effects related to a potential role in diabetic wound healing were displayed by *A. baetica.*

In the context of this research field, our findings are consistent with the growing body of evidence supporting the antidiabetic properties of various plant species, highlighting their potential as complementary therapeutic agents. However, these results should be interpreted with caution, as their biological relevance has not yet been confirmed in more complex biological systems. Although isolating and characterizing active compounds can help elucidate specific mechanisms, it is important to consider the potential synergistic effects among the constituents of the whole extracts, as observed particularly with *D. viscosa.* Further studies should focus on detailed chemical characterization of the plant extracts, with an emphasis on identifying and isolating the active compounds responsible for these biological effects. Subsequent in vivo testing of these isolated compounds and complete extracts would be essential to confirm their therapeutic potential and further validate their use in managing diabetes and related complications, such as wound healing. This study contributes to the expanding knowledge of plant-based therapies and their possible application in diabetes treatment, and future research could provide valuable insights into their clinical relevance.

## Figures and Tables

**Table 1:** Effectiveness of the aqueous extraction of the different plants analyzed expressed as extraction yield (%).

	*A. baetica*	*C. majus*	*D. viscosa*	*L. viridis*
Extraction yield (%)	12.17	10.79	16.75	12.13

**Table 2:** Results, expressed in concentration of the respective equivalents, obtained in the TPC, TFC, TAA, FRAP, and RP, and expressed in IC_50_, obtained in DPPH method, for the different aqueous extracts of the different plants analyzed.

	*A. baetica*	*C. majus*	*D. viscosa*	*L. viridis*
TPC (mg GAE/g dw)	118.80 ± 7.36 ^c^	76.51 ± 3.84 ^d^	477.10 ± 22.29 ^a^	282.98 ± 5.37 ^b^
TFC (mg QE/g dw)	25.28 ± 0.57 ^a^	3.16 ± 1.11 ^d^	22.87 ± 1.73 ^b^	16.99 ± 0.45 ^c^
TFC/TPC (%)	21.28	4.13	4.79	6.00
TAA (mg AAE/g dw)	193.30 ± 5.75 ^c^	121.55 ± 5.17 ^d^	704.54 ± 26.21 ^a^	561.30 ± 36.28 ^b^
FRAP (mg TE/g dw)	184.97 ± 4.84 ^c^	130.23 ± 2.53 ^d^	1132.99 ± 19.54 ^a^	785.45 ± 1.11 ^b^
RP (mg TE/g dw)	125.93 ± 4.21 ^c^	61.86 ± 2.77 ^d^	623.35 ± 24.02 ^a^	438.89 ± 17.94 ^b^
DPPH (µg/mL)	44.93 ± 3.26 ^b^	125.01 ± 8.84 ^a^	25.85 ± 0.75 ^d^	29.52 ± 0.75 ^c^

Note: All results are expressed as mean ± standard deviation (*n* = 3). Different superscript letters in the same row for the various samples in the same method (comparison of samples in the same method) indicate statistically significant differences between means as determined by Games–Howell post-hoc test following one-way ANOVA (with *p* < 0.05). TPC—total phenolic content; TFC—total flavonoid content; TAA—total antioxidant activity; FRAP—ferric reducing antioxidant power; RP—reductive potential; DPPH—free radical scavenging activity; GAE—gallic acid equivalents; QE—quercetin equivalents; AAE—ascorbic acid equivalents; TE—Trolox equivalents; dw—dry weight; TFC/TPC (%)—percentage contribution of total flavonoid content to total phenol content.

**Table 3:** Results of HPLC/PDA analysis expressed as concentration of flavonoids and phenolic acids in mg per 1 g of dried plant. All results are expressed as the mean associated with their standard deviation (n = 3).

	*A. baetica*	*C. majus*	*D. viscosa*	*L. viridis*
chlorogenic acid	0.05 ± 0	0.71 ± 0.03	0.13 ± 0	14.62 ± 0.15
isochlorogenic acid	<LOD	<LOD	<LOD	10.33 ± 0.06
caffeic acid	0.52 ± 0.75	0.02 ± 0	4.52 ± 0.94	13.22 ± 0.91
rutoside	2.09 ± 0.02	0.18 ± 0	<LOD	<LOD
protocatechuic acid	< LOD	<LOD	0.26 ± 0	0.14 ± 0
sinapic acid	<LOD	<LOD	1.98 ± 0.06	0.74 ± 0
isoquercetin	<LOD	<LOD	4.82 ± 0.01	<LOD
quercetin	<LOD	0.05 ± 0	0.06 ± 0.01	<LOD

<LOD—lower than the detection limit.

**Table 4:** Correlation matrix (Pearson correlation coefficients—*r*) of the results obtained through the different methods studied.

	TPC	TFC	TAA	FRAP	RP	DPPH	α-gluc	α-amyl	COL	ELA	LOX
TPC	1	0.4897	0.971 *	0.9869 *	0.9869 *	−0.717	−0.8396	−0.5165	0.8894	−0.1313	0.8495
TFC		1	0.4684	0.4307	0.4762	−0.8847	0.02067	−0.9799 *	0.07053	−0.8741	0.4748
TAA			1	0.9945 **	0.9968 **	−0.7711	−0.8726	−0.4481	0.823	−0.2027	0.6992
FRAP				1	0.9986 **	−0.7207	−0.8919	−0.4302	0.8755	−0.1254	0.756
RP					1	−0.7564	−0.8686	−0.4715	0.8516	−0.1777	0.7537
DPPH						1	0.3734	0.8155	−0.3202	0.7781	−0.4758
ɑ-gluc							1	−0.02284	−0.9132	−0.2727	−0.5632
ɑ-amyl								1	−0.1461	0.7748	−0.597
COL									1	0.3358	0.8234
ELA										1	0.007067
LOX											1

** Correlation is highly significant for a level of *p* < 0.001 (two-sided). * Correlation is significant at a *p* < 0.05 level (two-sided). TPC—total phenolic content; TFC—total flavonoid content; TAA—total antioxidant activity; FRAP—ferric reducing antioxidant power; RP—reducing potential; DPPH—free radical scavenging activity; ɑ-gluc—ɑ-glucosidase inhibition activity; α-amyl—α-amylase inhibition activity; COL—collagenase inhibition activity; ELA—elastase inhibition activity; LOX—lipoxygenase inhibition activity.

**Table 5:** Information regarding the place of harvest, date of harvest, and growing conditions of the plants used for the preparation of the aqueous extracts used in this work.

Plant	Harvest Location	Harvest Date	Growing Conditions
*Aristolochia baetica* L.	Rocha da Pena	May 2015	Wild plant
*Chelidonium majus* L.	Albufeira	March 2016	Cultivated plant
*Dittrichia viscosa* L.	Barranco do Velho	May 2015	Wild plant
*Lavandula virilis* L.	Barranco do Velho	May 2015	Wild plant

## Data Availability

Data are contained within the article and Supplementary Materials.

## Supplementary Materials

The following supporting information can be downloaded at: https://www.mdpi.com/article/10.3390/molecules30112432/s1, Figure S1: A. Chromatogram of *A. baetica* extract. B. Chromatogram of *C. majus* extract. C. Chromatogram of *D. viscosa* extract. D. Chromatogram of *L. viridis* extract.
