# Supplementary material for: Plant-Based Potential in Diabetes Management: In Vitro Antioxidant, Wound-Healing, and Enzyme Inhibitory Activities of Southern Algarve Species
Source: Molecules. 2025 Jun 1;30(11):2432. doi: 10.3390/molecules30112432 (PMC12156109; doi:10.3390/molecules30112432)
Supplement: Supplementary file 1 [file molecules-30-02432-s001.zip › molecules-3594738-supplementary.pdf]

Supplementary Material

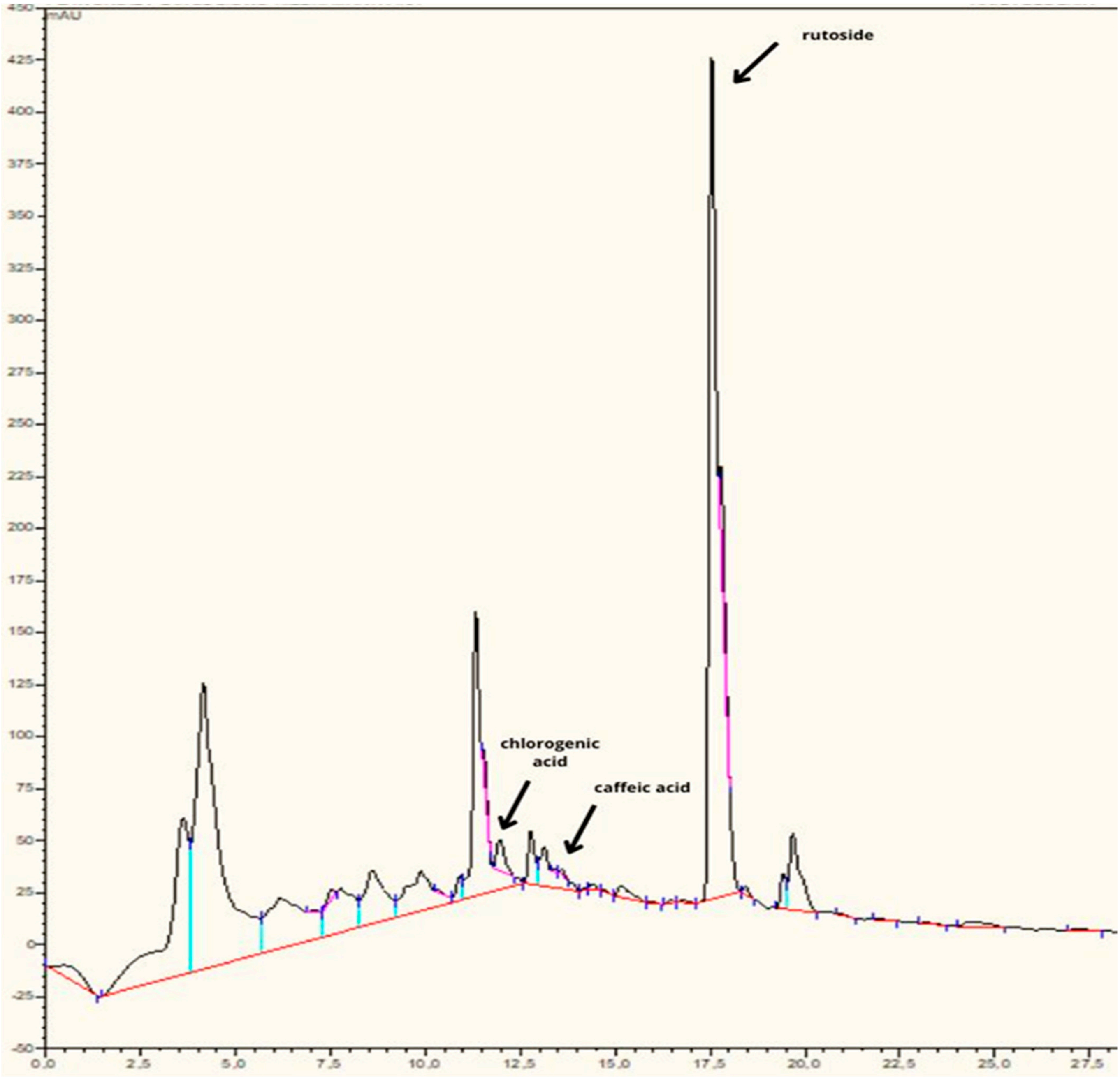

Figure S1. A. Chromatogram of *A. baetica* extract.

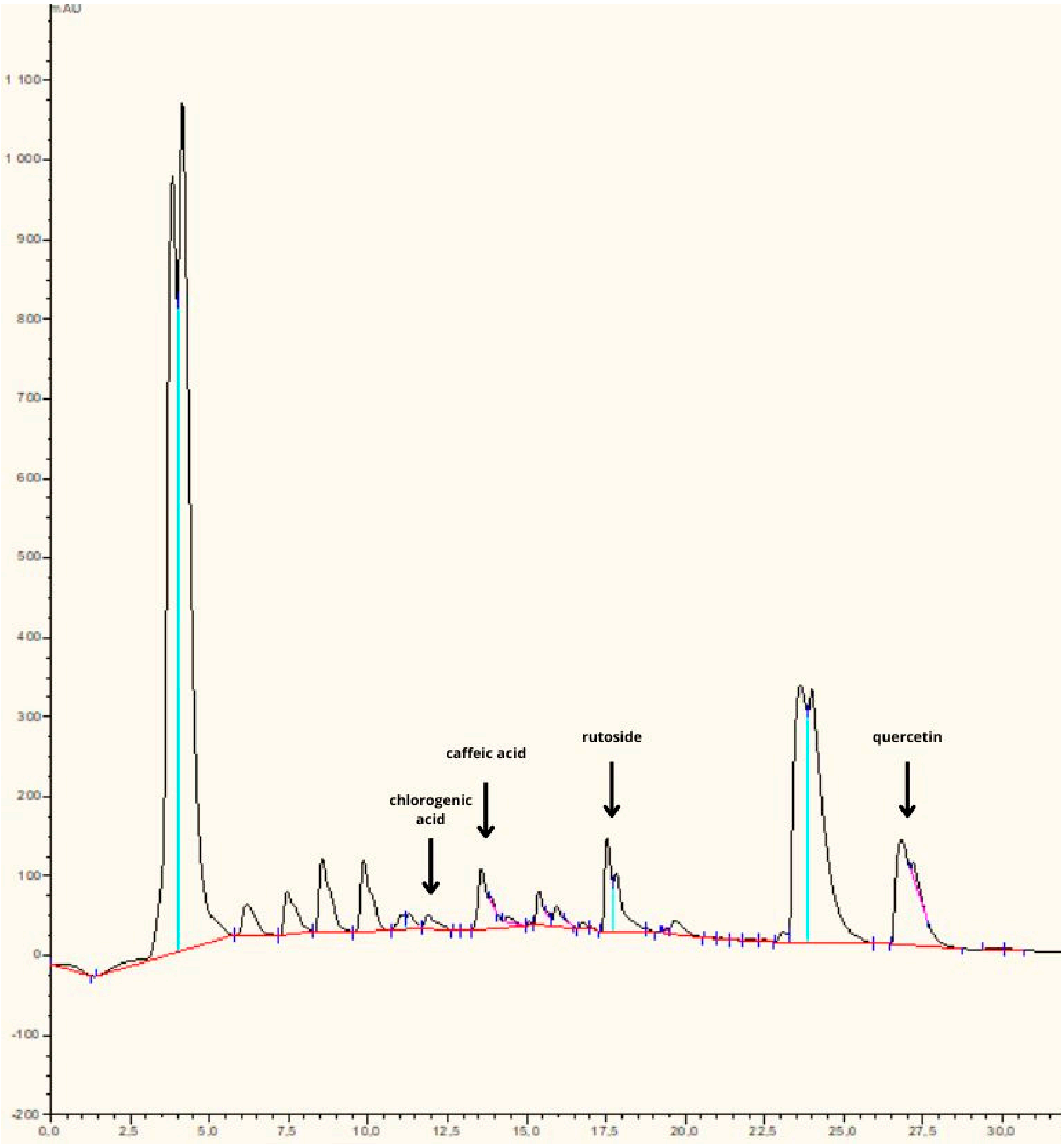

Figure S1. B. Chromatogram of *C. majus* extract.

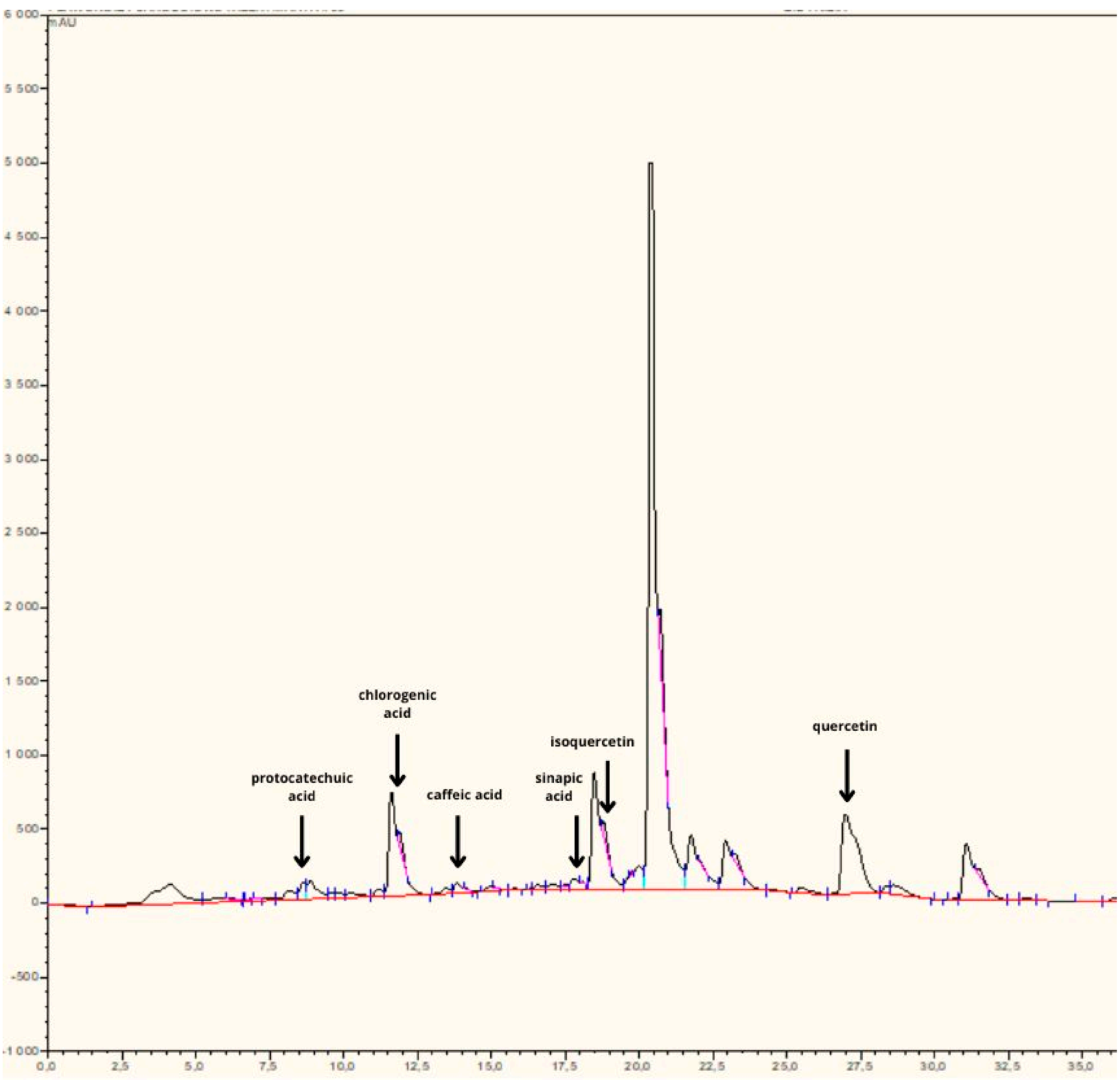

Figure S1. C. Chromatogram of *D. viscosa* extract.

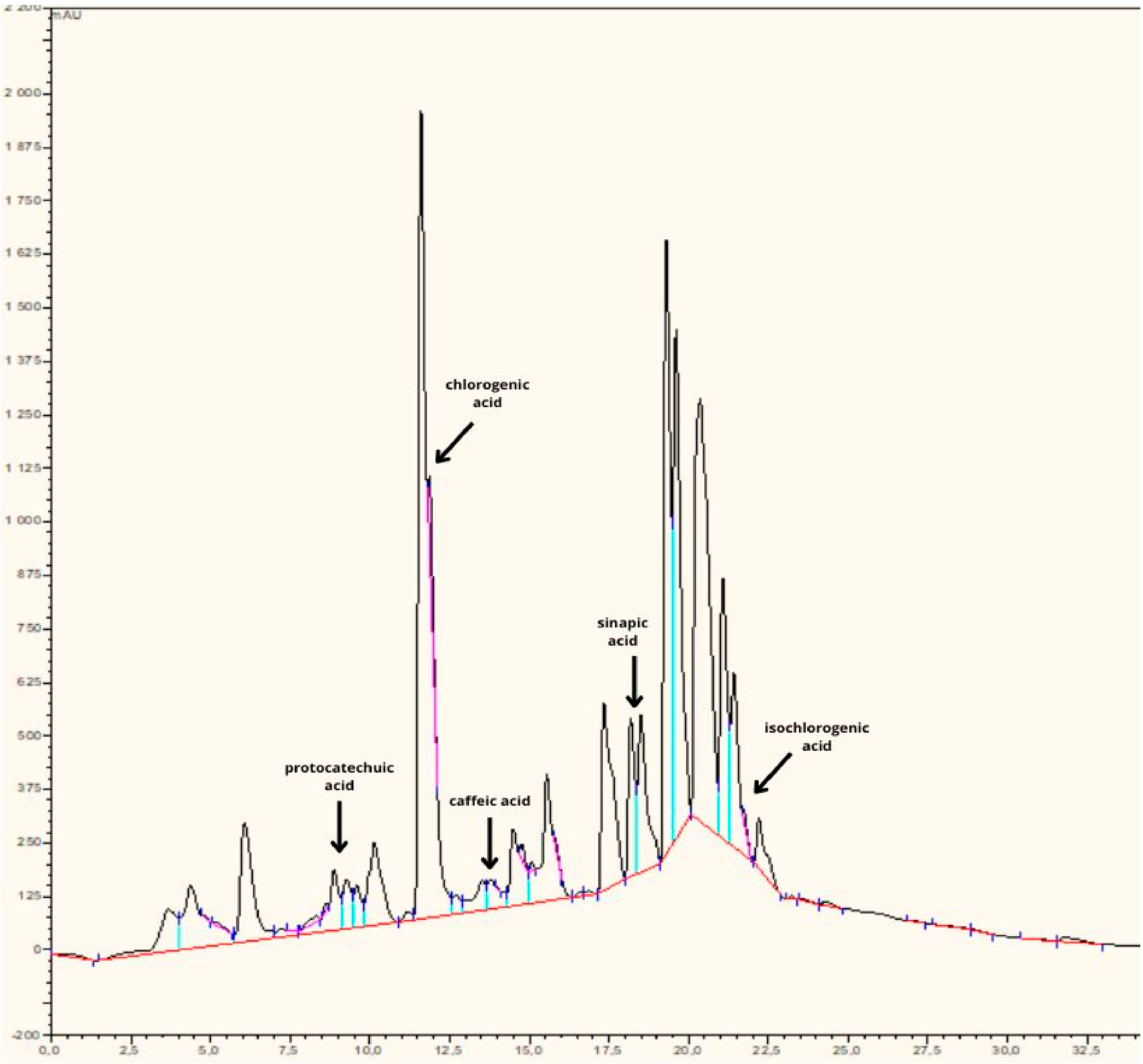

Figure S1. D. Chromatogram of *L. viridis* extract.
